# From Laboratory to Patient Access: A Scoping Review of the Multi-Dimensional Challenges in Drug Repurposing

**DOI:** 10.3390/pharmacy14030085

**Published:** 2026-06-11

**Authors:** Antonio Ivanov, Veselina Ruseva, Ines Hababa-Ivanova, Violeta Getova-Kolarova, Hristina Lebanova, Ilko Getov

**Affiliations:** 1 Department of Organization and Economics of Pharmacy, Faculty of Pharmacy, Medical University-Sofia, 2, Dunav Str., 1000 Sofia, Bulgaria; veselina.russeva@gmail.com (V.R.); ines.hababa98@gmail.com (I.H.-I.); v.getova@pharmfac.mu-sofia.bg (V.G.-K.); igetov@pharmfac.mu-sofia.bg (I.G.); 2 Department of Pharmaceutical Sciences and Social Pharmacy, Faculty of Pharmacy, Medical University-Pleven, 5800 Pleven, Bulgaria; hristina.lebanova@mu-pleven.bg

**Keywords:** drug repurposing, regulatory affairs, intellectual property, translational medicine, barriers to innovation, scoping review

## Abstract

Drug repurposing is often promoted as a faster, lower-risk alternative to de novo discovery, yet substantial barriers continue to limit successful implementation. We performed a scoping review of articles included in PubMed and ScienceDirect with the aim to identify and categorize challenges and analyze the intersections between them. Our review included 73 articles which revealed scientific, clinical, regulatory, economic, and implementation barriers, with the principal being the clinical translation of generated candidates. Scientific challenges include the necessity for new Phase II/III trials to validate efficacy, safety, and optimal dosing in new therapeutic contexts. Across disease areas, domain-specific barriers include subgroup-dependent responses in oncology, resistance and penetration challenges in anti-infectives, and data scarcity in rare diseases. Computational and AI-assisted approaches face fragmented data, model robustness, and insufficient validation. In addition, off-patent drugs face evidence requirements as rigorous as those for de novo entities, yet lack the market exclusivity incentives required to attract private investment. Additionally, an “institutional bottleneck” hinders academic researchers from bringing findings “on-label” due to a lack of regulatory infrastructure and collaborative frameworks. We conclude that drug repurposing requires a distinct translational paradigm involving multi-stakeholder collaboration and early regulatory engagement to bridge the gap between laboratory discovery and patient access.

## 1. Introduction

The traditional paradigm of de novo drug discovery is increasingly defined by its diminishing returns. Often referred to as “Eroom’s Law”—the functional inverse of Moore’s Law—the pharmaceutical industry has witnessed a consistent decline in the number of new drugs approved per billion dollars spent on R&D since the 1950s [1]. Currently, bringing a single new molecular entity (NME) to market requires an estimated 10 to 15 years and a capital investment ranging from $1 billion to over $2.6 billion [2]. In this climate of high attrition rates and escalating costs, drug repurposing—the process of identifying new medical indications for existing, approved, or investigational drugs—has emerged as a vital strategy for sustainable pharmaceutical innovation [3]. The primary allure of drug repurposing (also known as repositioning or redirecting) lies in its perceived efficiency. Because the approved candidate molecules have often already successfully passed Phase I safety trials, the risk of failure due to unacceptable toxicity is significantly mitigated [4]. Furthermore, the availability of existing pharmacokinetic data and established manufacturing supply chains can theoretically reduce the development timeline to 3–6 years and the associated costs to a fraction of traditional discovery [5]. From the repurposing of sildenafil for erectile dysfunction to the rapid deployment of dexamethasone during the COVID-19 pandemic, the history of pharmacy is punctuated by the profound clinical and commercial impact of repositioned therapies [6]. Despite these theoretical advantages, the transition “from laboratory to regulatory approval” is fraught with systemic complexity. While the scientific community frequently identifies high-potential repurposing candidates through advanced computational modeling and high-throughput screening, a significant “translation gap” persists [7]. This gap is not merely a result of biological uncertainty but is maintained by a fragmented ecosystem of scientific, clinical, practical, regulatory, and financial barriers. At the laboratory level, the “one drug, one target” philosophy is increasingly viewed as an oversimplification. Repurposed drugs often require different dosages or delivery mechanisms for new indications, necessitating new Phase II and III trials that carry similar risk of efficacy failure as NMEs [8]. Furthermore, the absence of robust biomarkers and the complexity of polypharmacology often obscure the exact mechanism of action in a new therapeutic context [9]. Perhaps the most daunting obstacles are found in the legal landscape. The regulatory framework in many jurisdictions is optimized for NMEs, leaving repurposed drugs—especially those that are off-patent or “orphaned”—in a state of legislative limbo [10]. Intellectual property rights present a particular paradox: while a new use for an old drug may be patentable, the “freedom to operate” is often hampered by existing patents, and the lack of strong data exclusivity period incentives often discourages private investment [11]. From a commercial perspective, the Return on Investment (ROI) for drug repurposing is frequently viewed as unfavorable compared to novel therapies. The phenomenon of “off-label” prescribing further complicates the business model. Once the new repurposed indication is approved for one drug product, all others with the same active substance can take advantage of this. Moreover, in the presence of generics, the cost of treatment can get lower, effectively cannibalizing the market for any company that invests in the formal repurposing approval process [12].

While existing literature has extensively covered individual aspects of drug repurposing—such as specific computational methods or legal analyses of patent law—there is a notable lack of integrated synthesis that maps the entire “end-to-end” journey of a repurposed molecule. The barriers are interconnected; for instance, a regulatory requirement for a new clinical trial (Scientific/Clinical) directly impacts the funding feasibility (Financial) and the eventual patent strategy (IP).

A scoping review is suitable for this topic as it allows for the “mapping” of a heterogeneous body of literature across multiple disciplines, including pharmacy, law, and economics [13]. By synthesizing these diverse perspectives, this review seeks to identify the specific bottlenecks that prevent laboratory breakthroughs from reaching clinical implementation and patient access.

### Objectives

This scoping review aims to:

- Identify and categorize the multi-dimensional challenges (scientific, clinical, regulatory, IP, and financial) currently discussed in the literature.
- Analyze the intersections between these barriers to understand how they compound one another.
- Examine how regulatory incentives, including patent protection, data protection, and market exclusivity, influence the feasibility of repurposing, investment decisions, and patient access.
- Highlight the current gaps in research and policy that must be addressed to streamline the drug-repurposing pipeline within the modern pharmacy ecosystem.

## 2. Materials and Methods

This scoping review was conducted to map the multi-dimensional barriers to drug repurposing. This review was performed in accordance with the Preferred Reporting Items for Systematic Reviews and Meta-Analyses extension for Scoping Reviews (PRISMA-ScR) guidelines [14]. The completed PRISMA-ScR checklist is available in the Supplementary Materials (Table S1).

A comprehensive literature search was performed in April 2026 across two primary electronic databases: PubMed and ScienceDirect. These databases were selected for their extensive coverage of biomedical, pharmaceutical, and interdisciplinary literature, ensuring the identification of challenges ranging from molecular chemistry to regulatory policies. To ensure this review reflects the current landscape—particularly in light of post-pandemic regulatory shifts and recent advancements in AI-driven discovery—the search was limited to articles published between January 2024 and December 2025.

The search string was designed to capture the core concepts of “drug repurposing” and the “barriers” associated with its implementation. The following Boolean search string was applied to titles, abstracts, and keywords:

(“drug repurposing” OR “drug repositioning” OR “drug reprofiling” OR “drug rescue”) AND (challenge OR obstacle OR barrier OR limitation)

Studies were assessed for inclusion based on the Population, Concept, and Context (PCC) framework.

Inclusion criteria:

- Studies focusing on any stage of the drug-repurposing pipeline (from discovery to clinical use).
- Articles discussing at least one of the following domains: scientific, clinical, practical, regulatory/IP, or financial/business challenges.
- Peer-reviewed original research and review articles.
- Publications in the English language and available via Open Access to facilitate transparency and data extraction.

Exclusion criteria:

- Editorials, commentaries, conference abstracts, and book chapters.
- Articles focusing solely on the repurposing of a specific drug/s without discussing the systemic barriers in front of the process.

The selection process was conducted in three distinct phases. First, the titles and abstracts of all identified records were screened independently by the authors against the eligibility criteria. Second, the full-text versions of all potentially relevant articles were retrieved and scrutinized for final inclusion. The third step included identifying the most influential and complete articles in each domain to be included in the discussion. Any disagreements between reviewers were resolved through a consensus discussion, or by consultation with a senior author if necessary.

Data from the included studies were extracted using a standardized charting form. The extracted data included:

Study Characteristics: Author(s) and year of publication.

Thematic Domains: Data were categorized into four pre-defined domains: (1) Scientific/Clinical, (2) Practical, (3) Regulatory/Intellectual Property, and (4) Financial/Business.

Nature of Barriers: Specific descriptions of the limitations identified.

Consistent with the scoping review methodology, no formal assessment of the methodological quality or “risk of bias” of the included studies was performed, as the primary objective was to map the extent of the literature rather than to provide a pooled estimate of effect [14,15]. The results are presented in a narrative format supported by tables and figures to illustrate the frequency and intersection of different barriers.

## 3. Results

**Figure 1:**
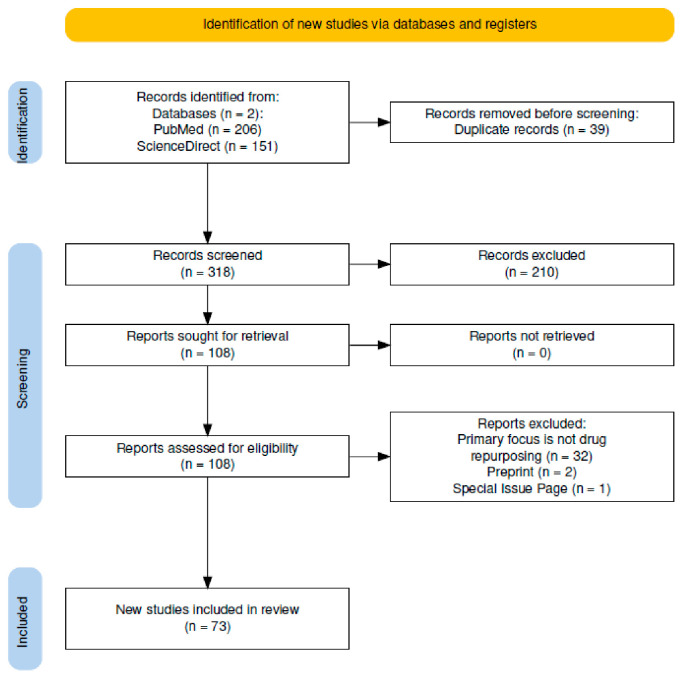
PRISMA flow diagram of studies included in this scoping review.

The database search identified 206 records from PubMed and 151 records from ScienceDirect. After removal of 39 duplicates, 318 records remained for title and abstract screening. Of these, 210 records were excluded as not meeting the eligibility criteria. The full texts of 108 articles were then assessed for eligibility, and 35 were excluded for reasons including primary focus not being drug repurposing, preprint manuscripts, and Special Issue pages. Ultimately, 73 studies met the inclusion criteria and were included in the final synthesis (Figure 1 and Table S2).

### 3.1. Articles’ Characteristics

The results show a clear increase toward 2025 publications (45/73), with fewer papers from 2024 (28/73), which suggests rising interest in the repurposing mechanism and possible post-pandemic and AI-enabled acceleration of efforts in this field. As regards country of origin of the projects, European countries represented the largest share of publications, accounting for approximately 44% of included studies, followed by North America (25%) and Asia (23%). In addition, high-income countries dominated, with the most represented being the United States, China, the United Kingdom, Italy and Germany, and moderate representation from the Netherlands, Spain, Belgium, Sweden and Saudi Arabia. In terms of article type, the collection was dominated by review-style papers, with a smaller set of original method-development or empirical studies. We have also observed the following patterns:

- 64% are single-country studies and 36% are multinational collaborations.
- European consortia were common in regulatory/policy studies.
- US/China dominated AI/computational studies.
- Disease-focused translational studies were more geographically diverse.
- Very low quantity of multinational projects (8%).

### 3.2. Therapeutic Focus

With respect to therapeutic focus, most included studies were disease-specific, comprising approximately two-thirds (66%) of the evidence base. These studies most commonly addressed oncology (18/73), neurodegenerative and neurological disorders (10/73), and infectious diseases and antimicrobial resistance (11/73), as well as a smaller number of studies focused on rare diseases (4/73) and cardiometabolic conditions (5/73). Oncology represented the most frequently covered therapeutic area, including studies on glioblastoma, gynecological malignancies, triple-negative breast cancer, tumor immunotherapy, and metabolic drug repurposing in cancer. Neurological and neurodegenerative indications included Alzheimer’s disease, amyotrophic lateral sclerosis, Rett syndrome, status epilepticus, and medulloblastoma. Anti-infective studies addressed bacterial resistance, antiviral repurposing, protozoan diseases, and antifungal resistance.

The remaining studies (25/73) had a general or platform-oriented therapeutic focus and addressed challenges applicable across multiple disease areas rather than a specific indication. These included computational drug-repurposing frameworks, graph-based and artificial intelligence methods, knowledge-graph and multi-omics approaches, as well as studies focused on regulatory, intellectual property, health technology assessment, and economic barriers. General-focus studies frequently addressed methodological or systemic barriers relevant to the broader repurposing ecosystem, rather than disease-specific translational challenges. Together, this distribution reflects a literature base weighted toward disease-oriented applications while also containing a substantial subset focused on cross-cutting methodological and structural barriers in drug repurposing.

### 3.3. Challenge Domains

Scientific/Clinical barriers were the most frequent overall (64/73) and the most common primary domain (41/73). Practical barriers were the next most common overall (45/73) and were frequently listed as the secondary domain (19/73). Regulatory/IP and Financial/Business barriers appeared less often overall (11/73 and 10/73, respectively), but they were important in the policy-oriented papers. The strongest co-occurrence was between Scientific/Clinical and Practical barriers—these two domains appeared together in 40 articles, reflecting a repeated pairing of translational limitations with implementation-related constraints. The next most common combined patterns linked validation/translational gaps with data or model limitations, and then with trial feasibility or heterogeneity/biomarker uncertainty. Regulatory/IP also clustered with Financial/Business barriers centered on exclusivity-based and access-related mechanisms, including limited exclusivity, data protection/exclusivity, ownership ambiguity, MAH dependency, formal approval barriers, and reimbursement-linked incentives.

### 3.4. Stage of Development

Among the original studies, most remained at the in silico/computational stage. One hybrid study integrated computational, in vitro, and real-world evidence and was therefore coded at the furthest stage reached, one study reached preclinical experimental testing, and one reached clinical testing. Overall, the evidence base was much richer in hypothesis-generation than in late-stage validation.

## 4. Discussion

### 4.1. Overview

Drug repurposing is increasingly framed not as a simple shortcut in drug development, but as a multifactorial and high-risk translational process. The central problem emerging from the evidence base is not a shortage of plausible candidates, but a recurrent mismatch between candidate generation and translational completion. Across various disease areas, the literature converges on the observation that repurposing may reduce early uncertainty surrounding prior human exposure, legacy safety data, and manufacturing readiness, but it does not eliminate the need to rebuild disease-specific evidence for efficacy, pharmacokinetics, patient selection, regulatory acceptability, and implementation [16,17,18]. Rather than removing development risk, repurposing often redistributes that risk away from discovery chemistry toward translational pharmacology, trial design, regulatory ownership, and commercial feasibility [17,19,20,21].

This is a critical interpretive point for the field. Drug repurposing is often described as uniformly faster and cheaper, yet the reviewed evidence supports a more qualified conclusion: repurposing is most efficient when the new indication is biologically proximate to the original one, when clinically relevant exposure is achievable without major reformulation or route changes, when endpoints are measurable, and when a sponsor or consortium can capture the value of a new label. When those conditions are absent, repurposing begins to resemble a partial restart of development rather than a shortcut [17,19,20,21]. This helps explain why high-throughput screening, network medicine, and AI-driven prioritization continue to generate more candidates than successfully licensed new indications. The principal challenge is therefore not discovering repurposing opportunities, but constructing translational systems capable of converting opportunities into approvable and reimbursable therapies.

### 4.2. Scientific and Clinical Domain—Demonstrating Efficacy and Safety as Primary Barrier

The included studies showed a clear and recurring emphasis on the need for indication-specific efficacy and safety evidence. Prior approval of a drug in one context was not presented in the literature as sufficient evidence for efficacy in a new indication. Instead, authors repeatedly described the need to reassess pharmacology, exposure, patient selection, and therapeutic response in the new disease setting. Oncology studies repeatedly showed that drugs developed for one receptor context or disease mechanism may require higher exposure, combination therapy, or altered delivery in the repurposed setting, at which point earlier safety assumptions may no longer hold [22]. Similar concerns arise in articles that describe antimicrobial and antiviral repurposing, where clinically active concentrations may be difficult to reach and changes in route or dosage may trigger new toxicological or early-phase requirements. Fluoxetine is a striking example, where antitumor effects emerge at doses far exceeding standard psychiatric use, effectively transforming an “old” drug into a new exposure problem [17]. This is particularly relevant in diseases with intrinsic barriers to tissue penetration, including medulloblastoma, Alzheimer’s disease, and chronic degenerative conditions such as osteoarthritis [22,23]. Tumor immunotherapy literature further sharpens this point further by showing that some repurposed agents demonstrate benefit only in defined patient subsets rather than across entire disease populations [22,23].

A second recurring theme was that repurposing often shifts, rather than removes, scientific uncertainty. Several studies highlighted situations in which a candidate appeared promising at the discovery stage but encountered barriers related to target relevance, tissue penetration, bioavailability, or heterogeneity of response. In such settings, the literature suggests that the repurposed medicine may require a level of evidence generation comparable to that needed for a new therapeutic use, even if the starting compound is already known. The reviewed studies therefore support a more nuanced interpretation of repurposing: prior human exposure may reduce some early uncertainties, but it does not eliminate the need for disease-specific pharmacological and clinical validation. Delivery remains a significant pharmacological hurdle, particularly for neurodegenerative diseases and brain tumors. Many candidates fail clinical translation because their molecular size or chemical composition prevents them from crossing the blood–brain barrier (BBB) to reach necessary therapeutic levels. While this can be overcome with formulation and administration changes such alterations effectively treat the compound as a “new drug” that must undergo Phase I clinical trials to assess safety in the new delivery context [24].

The literature also contains productive tensions. Some authors frame polypharmacology as an opportunity, others as a source of unpredictability. This contrast is visible in chronic thromboembolic pulmonary hypertension, where computational signals supported amiodarone analogues while real-world evidence associated amiodarone exposure with increased mortality [19,25]. These apparent contradictions suggest a shift from broad indication-level thinking toward stratified indication-with-subpopulation thinking. Repurposed drugs require renewed evaluation of mechanism, exposure, and responder populations rather than automatic transferability from the original therapeutic context [17,19,20,21].

Anti-infective repurposing provides the clearest example of class-specific challenges. In ESKAPE pathogens, many non-antibiotic molecules show activity only at concentrations outside realistic therapeutic windows, or fail due to biofilm penetration problems, efflux, and rapid metabolism [22]. Similar challenges arise in antiviral, antifungal and antimalarial repurposing, where resistance is not merely an implementation issue but a moving biological target [20,26,27,28].

Combination therapy may rescue a candidate biologically by increasing efficacy or suppressing resistance, yet simultaneously make it harder to develop institutionally. Every move toward combination therapy increases the burden of understanding interactions, formulation, IP, and regulatory pathways. Thus, the strategy most likely to save a repurposed candidate biologically may also make it harder to approve and reimburse [21,22].

### 4.3. The Practical Domain—Translating the Computational Results Using Fit-for-Purpose Clinical Trials

The reviewed literature also showed that different repurposing strategies generate different practical constraints. Target-based, phenotype-based, disease-oriented, and computational approaches each identify candidates under different assumptions and therefore require different forms of validation. Several studies emphasized that computational prioritization is useful for narrowing the search space, but that such methods remain dependent on downstream biological and clinical confirmation. In this sense, the practical domain functions as a bridge between discovery and implementation, and the evidence indicates that the bridge is often incomplete when model systems do not sufficiently reflect human pathophysiology [22,23].

The literature reveals a foundational tension between different repurposing strategies. Target-based approaches, while leveraging established pharmacological knowledge, are frequently criticized for their inherent bias toward existing targets, potentially overlooking novel therapeutic mechanisms. Evidence suggests these methods are further limited by the technical difficulty of covering membrane protein targets using current proteomic tools. In contrast, phenotype-driven approaches are viewed as more holistic but remain heavily dependent on the availability of physiologically relevant model systems that accurately reflect human disease—a major hurdle in complex conditions like Amyotrophic Lateral Sclerosis (ALS) [18].

Disease-oriented strategies, particularly those utilizing signature matching, face a “data-to-model” translation gap. While transcriptomic signatures provide a high-resolution snapshot of disease states, their utility is constrained by the limitations of model organisms and the secondary effects often found in post-mortem tissues, which may no longer contain the degenerated cells of interest [29,30]. Heterogeneous graph mining may miss higher-order biological context [31,32]; graph neural networks face robustness and label sparsity challenges [33]; and combination-oriented computational models introduce additional complexity when efficacy must be balanced against toxicity and interaction risk [34]. Published solutions such as hard-negative mining and multi-relational graph models suggest a rapidly maturing field while also exposing how fragmented the underlying data architecture remains [23,30,31,32,33,34,35,36,37].

Artificial intelligence and related data-driven methods were frequently described as enabling technologies, but the studies also consistently noted their limitations. AI-assisted methods show genuine potential in multi-omics integration, target identification, toxicity prediction, adaptive trial design, and literature-scale synthesis [23,25,31,32,33,34,35,36,37,38,39]. Yet, the same literature repeatedly identifies limiting conditions. The heterogeneity of omics datasets remains a significant hurdle to AI accuracy. Standardizing data formats is identified as a crucial step for the field to progress. here is a persistent lack of “explainable” workflows that move AI candidates beyond statistical association toward mechanistic validation. As noted in the study of amiodarone for CTEPH, even when a candidate stands out computationally, its ambiguous molecular mechanism complicates clinical prediction [40]. In addition, AI models are particularly susceptible to limited training data and overfitting when confronted with sparse input networks and The “black-box” nature of many algorithms creates a transparency gap that complicates regulatory acceptance and limits the openness of platforms for collaborative research. Finally, As AI paves the way for individualized treatment, there are still unresolved governance questions necessitating urgent need for frameworks that ensure data security and GDPR compliance without stifling the cross-border exchange of health data [39,40,41,42,43]. Collectively, this suggests AI should be viewed as a mean to compress search space and synthesize dispersed evidence, not as a substitute for mechanistic scrutiny or experimental validation [21,23].

The practical domain was characterized also by another recurring concern that promising repurposing signals do not automatically translate into feasible clinical development. Across the included studies, the practical challenge was not limited to trial logistics; rather, it encompassed endpoint selection, comparator choice, dose-finding, formulation changes, biomarker enrichment, and the feasibility of recruiting appropriate patient populations. This was particularly evident in rare diseases and pediatric indications, where limited sample sizes, long follow-up requirements, and endpoint uncertainty can make conventional trial designs difficult to implement. Across ALS, anti-infective development, rare diseases, pediatric CNS tumors, Alzheimer’s disease, and osteoarthritis, authors repeatedly question whether existing models capture relevant human pathophysiology, tissue kinetics, and response heterogeneity [18,20,21,22,23,25]. For rare diseases and specific pediatric oncology indications, the limited patient population makes traditional randomized controlled trials (RCTs) feasibility barriers, statistical underpower and costly execution. Furthermore, the data points to a “methodological gap” in how efficacy is measured. Many repurposed candidates aim for disease modification rather than acute symptom relief (e.g., in Alzheimer’s or Osteoarthritis), requiring long-term treatment durations that are difficult to fund without substantial industrial backing [20,21,23].

What emerges is a maturity map across disease areas. Oncology is rich in candidate generation but constrained by subgroup effects and combination logic. Anti-infectives are rich in mechanistic exploration but constrained by resistance biology and clinically meaningful potency. Rare diseases often depend on computational prioritization because classical evidence is sparse, but that makes validation even more important. In all three contexts, the deficit is less a lack of ingenuity than a lack of robust intermediate proof [16,22].

### 4.4. The Intersection Between Regulatory and Business Domains

One of the most durable obstacles is institutional fit. Regulatory pathways may exist, but they remain difficult to operationalize without coordinated engagement among regulators, MAHs, payers, and academia [16,22,23,41,43].

A recurring theme across the analyzed literature is the regulatory burden faced by repurposed medicines. Although repurposed drugs may benefit from existing knowledge of pharmacology, safety, and manufacturing, the extent of evidence required for authorization in a new indication remains context-dependent and may still be substantial. In practice, the evidentiary expectations vary according to the indication, the extent of formulation or route changes, the availability of prior clinical data, and the regulatory pathway used. Accordingly, the challenge is not that all off-patent drugs face identical requirements to de novo entities, but that repurposed products may still require a non-trivial, indication-specific evidence package despite already having a known active substance. This creates a tension between scientific feasibility and regulatory predictability, particularly when prior data cannot be fully leveraged because of jurisdictional differences in data protection rules and authorization pathways. This can result in a demand for tailored, high-cost clinical trials to prove efficacy in new indications which combined with the lack of financial incentives can offer limited commercial profitability and a low return on investment (ROI) [16,42].

The evidence suggests that investment decisions are shaped by a combination of factors rather than by market exclusivity alone. The absence or weakness of market exclusivity can discourage pharmaceutical investment, but the extent of this effect depends on other determinants, including data protection, patent scope, expected development costs, indication size, reimbursement prospects, and the likelihood of recouping investment in the presence of generic competition. In this regard, repurposing may be commercially unattractive not solely because exclusivity is limited, but because the overall incentive structure is often misaligned with the regulatory and economic effort required to obtain a new indication. For instance, “method-of-use” patents, which are often the only remaining intellectual property (IP) protection for repurposed drugs, are described as “weak” and difficult to enforce, as generics can easily be prescribed “off-label” for the new condition [44,45].

A critical gap identified in the data is the “Institutional Bottleneck.” While academic institutions and non-profit organizations are more willing to explore repurposing options for medicines out of protection, they fundamentally lack the regulatory infrastructure to bring an indication “on-label”. Bringing a drug on-label requires identifying and collaborating with the Marketing Authorization Holder (MAH) of the finished product, a task academic organizations often find difficult due to a lack of expertise and resources. Furthermore, researchers highlight a significant “translational disconnect”: academics often focus on generating preclinical evidence but neglect the regulatory hurdles and market access challenges, leading to “pointless studies” that fail to reach the clinic. There is an urgent call across multiple papers for a collaborative framework involving not-for-profit organizations, industry, patient groups, and HTA bodies to facilitate the on-label transition [46,47].

### 4.5. Strengths and Limitations of This Review

This review has several methodological and conceptual strengths. First, it provides an integrative synthesis of drug-repurposing barriers across the full translational continuum, spanning scientific, clinical, practical, regulatory, intellectual property, and economic dimensions. In contrast to analyses that treat these domains independently, this review emphasizes their interdependence and conceptualizes translational failure as an emergent property of interacting barriers rather than isolated deficiencies.

Several limitations should also be acknowledged. The search strategy was restricted to PubMed and ScienceDirect and limited to English-language open-access publications, which may have excluded relevant evidence from other databases, grey literature, or jurisdiction-specific regulatory sources (particularly those focusing on regulatory science, health policy, intellectual property, and pharmacoeconomics). Second, this review was limited to English-language publications, potentially introducing language bias and underrepresenting evidence generated in non-English-speaking jurisdictions.

Another important limitation is that only open-access publications were eligible for inclusion. This criterion was adopted to facilitate transparent screening, full-text assessment, and reproducible data extraction. However, it may have introduced selection bias by excluding relevant subscription-based literature, including potentially influential publications in regulatory affairs, pharmaceutical policy, intellectual property law, health technology assessment, and health economics. Consequently, some perspectives on the regulatory and commercial dimensions of drug repurposing may be underrepresented in the present synthesis.

As consistent with scoping review methodology, no formal appraisal of methodological quality or risk of bias was undertaken and the interpretive nature of thematic synthesis introduces a degree of subjectivity, although structured screening and consensus procedures were used to mitigate this. The objective of this review was to map the breadth and nature of the available literature rather than to evaluate the methodological quality of individual studies or generate pooled estimates of effect. Therefore, the findings should be interpreted as a synthesis of reported barriers and challenges rather than as evidence of their relative magnitude or causal importance.

In addition, the evidence base was unevenly distributed across domains. Scientific and clinical studies constituted the majority of included publications, whereas regulatory, intellectual property, and business-oriented studies were less frequently represented. This imbalance reflects the current state of the literature but may influence the relative prominence of themes identified during synthesis. Finally, the temporal restriction to recent publications was intended to capture the contemporary post-pandemic and AI-enabled landscape of drug repurposing. However, this approach may have excluded earlier foundational studies that continue to influence current regulatory and translational practices. On the other hand, due to the rapid evolution of regulatory frameworks, computational methodologies, and translational infrastructures, some conclusions may require reassessment as the field develops.

## 5. Conclusions

Drug repurposing remains a highly promising strategy for accelerating therapeutic innovation. However, our scoping review shows that its main barriers are systemic rather than merely technical. The central challenge is not the identification of candidate molecules, but the difficulty of translating promising candidates into therapies that are clinically validated, regulatorily acceptable, commercially viable, and accessible to patients. Across the scientific, clinical, practical, regulatory, intellectual property, and financial domains, the barriers identified in this review interact cumulatively, creating a persistent translational bottleneck between laboratory discovery and real-world implementation.

The evidence indicates that repurposed medicines often behave as new therapeutic entities in their new indications, requiring renewed evaluation of efficacy, benefit–risk ratio, dosing, formulation, patient selection, and clinical utility. Computational approaches, network medicine, and artificial intelligence have substantially expanded the capacity to generate repurposing hypotheses, but they do not resolve downstream requirements for mechanistic validation, robust preclinical models, biomarker-guided clinical trials, regulatory ownership, and reimbursement pathways. Similarly, current legal and economic frameworks often fail to align public-health value with commercial incentives, particularly for off-patent and generic medicines.

Taken together, these findings suggest that drug repurposing should not be viewed primarily as a shortcut around conventional drug development. Rather, it should be understood as a distinct translational paradigm that requires coordinated evidence generation, early regulatory and payer engagement, clear intellectual property strategies, and multi-stakeholder collaboration. A central consideration in this process is the benefit–risk evaluation of the repurposed use: prior approval in one indication does not eliminate the need to re-assess efficacy, safety, dosing, formulation, patient selection, and clinical utility in the new therapeutic context. Future progress will depend less on generating additional candidate lists and more on closing the structural gap between discovery, authorization, reimbursement, and clinical adoption. Bridging this gap will determine whether drug repurposing remains an opportunistic strategy or evolves into a mature, scalable, and sustainable model for pharmaceutical innovation.

## 6. Future Directions

**Figure 2:**
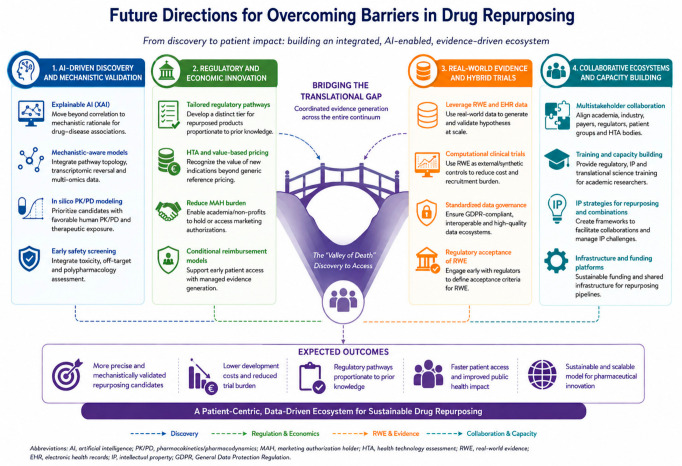
Future directions for overcoming barriers in drug repurposing.

The findings of this scoping review indicate that future progress in drug repurposing will depend less on the generation of additional candidate lists and more on the development of translational systems capable of converting promising signals into validated, approvable, and implementable therapies. The reviewed literature consistently shows that the major bottleneck is not candidate identification alone, but the transition from computational or preclinical promise to indication-specific evidence, regulatory acceptability, and patient access. This is reflected in the predominance of scientific/clinical and practical barriers, together with the repeated co-occurrence of these domains across the included studies (Figure 2).

While computational pipelines have significantly accelerated candidate generation, a primary future requirement is the implementation of Explainable AI (XAI). Future discovery strategies must move beyond statistical drug-disease associations (DDAs) toward workflows that provide a transparent pharmacological rationale. There is also an urgent need for “mechanistic-aware” algorithms that integrate pathway topology with transcriptomic reversal data to predict not just that a drug might work, but how it achieves therapeutic efficacy in a new tissue context. Integrating In Silico PK/PD modeling into early-stage AI discovery will be vital to identify candidates that can reach therapeutic concentrations at safe, human-equivalent doses before costly clinical trials begin.

The “regulatory-economic paradox” identified in this review—where off-patent drugs face the same evidence requirements as de novo entities—demands a new legislative tier. Future policy research should focus on creating a predictable and tailored Health Technology Assessment (HTA) process for off-patent repurposed medicines. This framework should move away from generic-based pricing and instead recognize the value of the “new” indication, perhaps through value-based pricing or conditional reimbursement models. To address the “MAH burden”, legislative efforts must empower academic institutions and non-profit organizations to hold Marketing Authorizations (MAs) or create platforms that facilitate low-cost partnerships between academia and generic manufacturers.

As evidenced by projects like MEDI-X and DeepDRA, the future of repurposing validation lies in the use of Real-World Evidence (RWE) derived from Electronic Health Records (EHR) and claim data. The development of the European Health Data Space (EHDS) offers a future opportunity to perform “computational clinical trials” at scale. However, this requires standardized data governance and robust GDPR-compliant frameworks to ensure that AI-driven RWE can be accepted as “pivotal” evidence by agencies like the EMA or FDA. We anticipate a shift toward hybrid clinical trial designs that use RWE as synthetic control arms, significantly reducing the cost and recruitment burden for rare disease repurposing.

The “Institutional Gap” can only be closed through the creation of specialized ecosystems that provide technical and regulatory training to academic researchers. Future initiatives should mirror the REMEDi4ALL approach, providing “repurposing bootcamps” for academics to learn the complexities of drug formulation, IP strategy, and regulatory submission. Future research must address the “IP minefield” of drug–drug combinations. Creating pre-competitive frameworks where different Marketing Authorization Holders (MAHs) can collaborate on synergistic combinations (e.g., in oncology or antimicrobial resistance) without fear of patent infringement is essential for modern precision medicine.

In summary, the future of drug repurposing in pharmacy practice is not merely about finding “new uses for old drugs,” but about building a sustainable, data-driven, and patient-centric ecosystem. By addressing the current legislative stagnation and focusing on mechanistic AI validation, the field can finally deliver on its promise of providing affordable, effective therapies for high-burden and rare diseases.

## Data Availability

No new data were created or analyzed in this study. Data sharing is not applicable to this article.

## Supplementary Materials

The following supporting information can be downloaded at: https://www.mdpi.com/article/10.3390/pharmacy14030085/s1, Table S1: PRISMA ScR Checklist; Table S2: Table of results included in this scoping review. (References [48,49,50,51,52,53,54,55,56,57,58,59,60,61,62,63,64,65,66,67,68,69,70,71,72,73,74,75,76,77,78,79,80,81,82,83,84,85,86,87] are cited in the Supplementary Materials).

## Abbreviations

The following abbreviations are used in this manuscript:

NME	New molecular entity
ROI	Return on Investment
IP	Intellectual Property
