# Supplementary material for: From Laboratory to Patient Access: A Scoping Review of the Multi-Dimensional Challenges in Drug Repurposing
Source: Pharmacy (Basel). 2026 Jun 11;14(3):85. doi: 10.3390/pharmacy14030085 (PMC13306824; doi:10.3390/pharmacy14030085)
Supplement: Supplementary file 1 [file pharmacy-14-00085-s001.zip › Table S2 - Table of results included in the scoping review-edited.pdf]

Table of results included in the scoping review

No.	Article	Reference Number	Objective	Key barriers/challenges identified	Main conclusion/contribution	Primary Domain; Secondary Domain(s)
1.	Bae WH et al. 2024. *Challenges and advances in glioblastoma targeted therapy: the promise of drug repurposing and biomarker exploration*	48	To review advances in glioblastoma targeted therapy, with attention to repurposing and biomarker-based approaches.	Blood–brain barrier penetration, tumour heterogeneity, limited predictive value of models, biomarker uncertainty, poor clinical translation.	Glioblastoma repurposing requires biomarker-guided selection, CNS delivery strategies and stronger translational validation before clinical implementation.	Scientific/Clinical; Practical
2.	Fuchs C et al. 2024. *Drug repurposing in Rett and Rett-like syndromes: a promising yet underrated opportunity?*	49	To assess the potential of repurposing in Rett and Rett-like syndromes.	Rare-disease evidence scarcity, small patient populations, limited clinical trials, weak translational infrastructure.	Repurposing may be valuable in rare neurodevelopmental disorders, but requires structured evidence generation and feasible trial designs.	Scientific/Clinical; Practical
3.	Walker MC. 2024. *Drug repurposing in status epilepticus*	45	To examine repurposed therapeutic options for status epilepticus.	Acute-trial feasibility, dose optimization, limited efficacy evidence, safety uncertainty in emergency settings.	Prior approval does not remove the need for indication-specific validation in acute neurological conditions.	Scientific/Clinical; Practical
4.	Avolio E et al. 2025. *Shared molecular, cellular, and environmental hallmarks in cardiovascular disease and cancer: Any place for drug repurposing?*	47	To explore shared mechanisms between cardiovascular disease and cancer as a basis for repurposing.	Mechanistic transferability uncertainty, biological heterogeneity, disease-context specificity.	Shared pathobiology can generate repurposing hypotheses, but disease-specific validation remains essential.	Scientific/Clinical
5.	Dong X et al. 2024. *Emerging technologies for drug repurposing: Harnessing the potential of text and graph embedding approaches*	37	To review text- and graph-embedding methods for drug repurposing.	Data fragmentation, incomplete biomedical knowledge graphs, model interpretability, validation gaps.	Embedding approaches improve candidate prioritization but remain hypothesis-generating tools requiring external validation.	Practical; Scientific/Clinical
6.	Xia Y et al. 2024. *Drug repurposing for cancer therapy*	50	To review drug repurposing opportunities in cancer therapy.	Tumour heterogeneity, exposure/dosing uncertainty, resistance, limited clinical validation.	Oncology repurposing is promising but depends on stratified development, dose optimization and robust clinical evidence.	Scientific/Clinical

7.	Carroll E et al. 2025. *Drug repurposing in amyotrophic lateral sclerosis (ALS)*	18	To review drug repurposing strategies in ALS.	Weak disease models, limited intermediate evidence, heterogeneous disease progression, trial endpoint challenges.	ALS repurposing is constrained less by candidate scarcity than by poor translational predictivity and difficult trial design.	Scientific/Clinical; Practical
8.	Qi T et al. 2024. *From genetic associations to genes: methods, applications, and challenges*	51	To review methods translating genetic associations into candidate genes.	Target prioritization uncertainty, causal inference limitations, incomplete functional validation.	Genetic evidence can support repurposing target discovery, but requires functional and disease-context validation.	Scientific/Clinical; Practical
9.	Cousins HC et al. 2024. *Computational Approaches to Drug Repurposing: Methods, Challenges, and Opportunities*	52	To review computational methods for drug repurposing.	Data quality, model robustness, reproducibility, validation and generalizability limitations.	Computational approaches accelerate candidate identification but cannot replace experimental and clinical validation.	Practical; Scientific/Clinical
10	Li Y et al. 2025. *Drug Repurposing: An Emerging Strategy in Staphylococcus aureus Infections*	53	To evaluate repurposing strategies against *Staphylococcus aureus*.	Resistance, biofilm penetration, PK/PD constraints, toxicity at antibacterial concentrations.	Repurposing for *S. aureus*requires clinically achievable exposure and resistance-aware validation.	Scientific/Clinical
11	Tuci S et al. 2025. *Antiviral drug repurposing: different approaches and the case of antifungal drugs*	44	To review antiviral repurposing strategies, including antifungal drugs as antiviral candidates.	Dose escalation, altered routes, uncertain antiviral efficacy, renewed toxicity concerns.	Antiviral repurposing often recreates early-development challenges when new exposure profiles are required.	Scientific/Clinical; Practical
12	Malla R et al. 2024. *Revitalizing Cancer Treatment: Exploring the Role of Drug Repurposing*		To review the role of drug repurposing in cancer treatment.	Subgroup-specific efficacy, combination complexity, biomarker limitations.	Cancer repurposing may expand therapeutic options but requires precision-guided clinical evaluation.	Scientific/Clinical
13	Kadasah SF et al. 2024. *Beyond Psychotropic: Potential Repurposing of Fluoxetine toward Cancer Therapy*	17	To assess fluoxetine as a potential anticancer repurposing candidate.	Dose/exposure differences, uncertain anticancer therapeutic window, toxicity concerns.	Fluoxetine illustrates how a familiar drug may behave like a new therapeutic entity in a repurposed oncology setting.	Scientific/Clinical

14	Weth FR et al. 2024. *Unlocking hidden potential: advancements, approaches, and obstacles in repurposing drugs for cancer therapy*	54	To review advances and barriers in cancer drug repurposing.	Tumour heterogeneity, incomplete mechanism validation, trial design barriers.	Successful oncology repurposing requires mechanism-based selection and rigorous clinical testing.	Scientific/Clinical
15	Alghalayini A et al. 2025. *Drug repurposing in gynaecological cancers: Proven drugs for new challenges*	16	To evaluate repurposed drugs for gynaecological cancers.	Biomarker uncertainty, patient selection, efficacy validation, regulatory translation.	Repurposing in gynaecological cancer requires disease-specific evidence and regulatory planning.	Scientific/Clinical; Regulatory/IP
16	Salvati A et al. 2025. *Multi-omics based and AI-driven drug repositioning for epigenetic therapy in female malignancies*	25	To explore AI and multi-omics drug repositioning for epigenetic therapy.	Data integration, interoperability, validation, governance and model robustness.	AI and multi-omics broaden discovery but require validated, transparent and interoperable pipelines.	Practical; Scientific/Clinical
17	Dong J et al. 2025. *Drug functional remapping: a new promise for tumor immunotherapy*	55	To examine functional remapping of drugs for tumour immunotherapy.	Subpopulation-specific benefit, mechanistic complexity, combination uncertainty.	Immunotherapy repurposing may be valuable but must be stratified by tumour and patient characteristics.	Scientific/Clinical
18	Elechi KW et al. 2025. *Translational Success and Pharmacoeconomic Lessons of Pandemic-Driven Drug Repurposing*	56	To analyse translational and pharmacoeconomic lessons from pandemic repurposing.	Emergency evidence standards, reimbursement uncertainty, crisis-dependent incentives.	Pandemic repurposing shows acceleration is possible, but success may depend on exceptional policy and funding conditions.	Financial/Business; Practical
19	Steiert D et al. 2024. *An exploration into CTEPH medications: Combining NLP, embedding learning, in vitro models, and real-world evidence for drug repurposing*	40	To integrate computational, experimental and real-world evidence for CTEPH repurposing.	Discordance between computational predictions and real-world evidence, safety signals, validation needs.	Multi-source evidence improves prioritization but must be reconciled before clinical translation.	Scientific/Clinical; Practical
20	Abdel-Rasol M et al. 2025. *Advancing Medulloblastoma Treatment: Molecular Mechanisms, Drug*	57	To review molecular mechanisms and repurposing strategies in medulloblastoma.	Paediatric evidence gaps, CNS penetration, molecular heterogeneity, trial feasibility.	Medulloblastoma repurposing requires precision therapy approaches and CNS-relevant validation.	Scientific/Clinical; Practical

	Repurposing, and Precision Therapies					
21	Guo S et al. 2025. *Drug repurposing against drug-resistant ESKAPE pathogens*	22	To review repurposing against resistant ESKAPE pathogens, with focus on *Pseudomonas aeruginosa*.	Biofilms, resistance mechanisms, efflux, clinically meaningful potency.	Anti-ESKAPE repurposing must account for pathogen-specific resistance and exposure constraints.	Scientific/Clinical
22	Cui H et al. 2024/2025. *Heterogeneous graph contrastive learning with gradient balance for drug repositioning*	23	To develop a heterogeneous graph contrastive learning model for drug repositioning.	Label sparsity, robustness, external validation, biological interpretability.	Graph learning improves prediction but remains a prioritization method requiring validation.	Practical; Scientific/Clinical
23	Li X et al. 2024. *Integrated edge information and pathway topology for drug-disease associations*	32	To improve drug–disease association prediction through edge and pathway information.	Incomplete biological context, representation fidelity, validation requirements.	Pathway-aware models may improve signal detection but do not establish clinical utility alone.	Practical; Scientific/Clinical
24	Salgueiro MJ et al. 2025. *Nanotechnology-Driven Repurposing in Radiopharmacy*	58	To discuss nanotechnology-driven repurposing in radiopharmacy beyond oncology.	Delivery, formulation, safety assessment, regulatory complexity.	Nano-enabled repurposing may expand utility but introduces new development and regulatory requirements.	Practical; Regulatory/IP
25	Tshimweneka V et al. 2025. *Old Drugs, New Battles: Repurposed Drug Classes in Triple-Negative Breast Cancer*	59	To review repurposed drug classes in triple-negative breast cancer.	Aggressive disease biology, combination therapy complexity, patient stratification.	Repurposed classes may support TNBC therapy but require combination-aware, biomarker-guided validation.	Scientific/Clinical
26	Hetta HF et al. 2025. *Beyond Conventional Antifungals: Combating Resistance Through Novel Therapeutic Pathways*	27	To review novel antifungal pathways and resistance strategies.	Antifungal resistance, limited drug classes, toxicity, interactions, tissue exposure.	Antifungal repurposing must address resistance and interaction risks alongside efficacy.	Scientific/Clinical
27	Gonzalez Gomez et al. 2024. *Optimizing in silico drug discovery: simulation of*	29	To benchmark in silico discovery using simulated connected	Benchmark quality, disease-signature fidelity, reproducibility, validation.	Signature-based repurposing depends on reliable benchmark systems and biologically meaningful expression data.	Practical; Scientific/Clinical

	connected differential expression signatures		differential expression signatures.			
28	Wang Z et al. 2025. *AI for antimicrobial resistance: bibliometric and visualization analysis*	60	To map knowledge trends in AI for antimicrobial resistance.	Evidence fragmentation, model validation, clinical translation of AI outputs.	AI is expanding in AMR research but requires translational and clinical integration.	Practical; Scientific/Clinical
29	Huang LC et al. 2025. *DrugReX: an explainable drug repurposing system powered by LLMs and knowledge graph*	39	To develop an explainable LLM- and knowledge graph-based drug repurposing system.	Explainability, reliability, knowledge completeness, validation.	Explainable AI may improve trust in prioritization but still requires experimental validation.	Practical; Scientific/Clinical
30	Garcia-Diaz M et al. 2025. *Overcoming barriers to off-patent drug repurposing: lifecycle-based policy solutions*	41	To propose lifecycle-based policy solutions for off-patent repurposing.	Weak incentives, ownership ambiguity, limited exclusivity, regulatory and reimbursement gaps.	Off-patent repurposing requires policy reform and lifecycle-based incentive mechanisms.	Regulatory/IP; Financial/Business
31	Del Álamo M et al. 2024. *Funding multinational investigator-initiated clinical studies in Europe*	46	To analyse funding and coordination of multinational investigator-initiated studies.	Funding fragmentation, administrative burden, cross-border regulatory complexity, limited infrastructure.	Academic-led repurposing requires sustainable funding and multinational trial infrastructure.	Financial/Business; Practical; Regulatory/IP
32	Chen C et al. 2025. *MRDDA: a multi-relational graph neural network for drug–disease association prediction*	35	To develop a multi-relational graph neural network for drug–disease association prediction.	Data sparsity, representation fidelity, external validation.	Multi-relational models enhance prediction but remain exploratory without biological validation.	Practical; Scientific/Clinical
33	Khan SA et al. 2025/2026. *Mechanisms to medicines: navigating drug repurposing strategies in Alzheimer's disease*	24	To review repurposing strategies in Alzheimer's disease.	Disease heterogeneity, BBB penetration, biomarker limitations, long-duration trials.	Alzheimer's repurposing requires better patient stratification, delivery systems and biomarker-guided evidence.	Scientific/Clinical; Practical
34	Voloudakis G et al. 2025. *Genetically based computational drug repurposing framework for COVID-19*	61	To apply a genetics-based computational framework to identify COVID-19 candidates.	Genetic-to-drug translation uncertainty, validation requirements, emergency evidence limitations.	Genetic frameworks can accelerate candidate discovery but require clinical validation.	Practical; Scientific/Clinical

35	van der Walt M et al. 2025. *Reverse pharmacophore mapping of istradefylline for major depressive disorder*	62	To propose a reverse pharmacophore workflow for repurposing istradefylline.	Mechanistic uncertainty, hypothesis validation, computational-to-clinical translation.	Reverse pharmacophore mapping supports low-cost hypothesis generation but does not replace experimental validation.	Practical; Scientific/Clinical
36	Yin H et al. 2025. *Unlocking biological insights from differentially expressed genes*	31	To review concepts and methods for interpreting differentially expressed genes.	Causal inference, pathway interpretation, false-positive biological signals.	DEG analysis can support target discovery but needs careful causal and functional validation.	Scientific/Clinical; Practical
37	Luconi M et al. 2025. *Repurposing glucose-lowering drugs for cancer therapy*	42	To assess glucose-lowering drugs as potential cancer therapies.	Heterogeneous clinical evidence, patient selection, reimbursement and commercial uncertainty.	Metabolic drug repurposing in cancer is promising but requires stratified clinical validation and incentive alignment.	Scientific/Clinical; Financial/Business
38	Kumar S et al. 2025. *Current strategies against multidrug-resistant Staphylococcus aureus*	63	To review current and future strategies against MDR *S. aureus*.	Resistance, biofilm, toxicity, limited durable treatment options.	MDR bacterial repurposing requires resistance-aware strategies and clinically meaningful efficacy.	Scientific/Clinical
39	van Houten P et al. 2024. *Digoxin treatment does not reinduce radioiodine uptake in thyroid carcinoma*	64	To test whether digoxin can restore radioiodine uptake in refractory thyroid carcinoma.	Failure of mechanistic translation, limited clinical effect, disease-specific non-response.	Negative findings illustrate that mechanistic rationale alone is insufficient for repurposing success.	Scientific/Clinical
40	Theodoridis L et al. 2025. *Antimalarial drug resistance and drug discovery*	20	To review antimalarial resistance and future drug discovery.	Resistance evolution, partner-drug dynamics, parasite adaptation.	Antimalarial repurposing must account for resistance biology and durability of effect.	Scientific/Clinical
41	Tenchov R et al. 2024. *Alzheimer's Disease: Exploring the Landscape of Cognitive Decline*	65	To review Alzheimer's disease pathophysiology and therapeutic landscape.	Multifactorial pathology, biomarker uncertainty, long-term outcome assessment.	Alzheimer's disease requires mechanism-specific and biomarker-supported repurposing strategies.	Scientific/Clinical
42	Papareddy P et al. 2025. *Rediscovering nitroxoline: a metal-chelating agent bridging infection and cancer*	19	To examine nitroxoline as a repurposed agent across infection and cancer.	Polypharmacology, benefit–risk reassessment, cross-indication mechanism uncertainty.	Nitroxoline illustrates the dual promise and complexity of polypharmacological repurposing.	Scientific/Clinical
43	Paul JK et al. 2025. *Harnessing machine learning for improved*	66	To review machine learning across	Data quality, interpretability,	Machine learning may support discovery and care pathways but requires validation and governance.	Practical; Scientific/Clinical

	diagnosis, drug discovery, and patient care		diagnosis, discovery and patient care.	implementation readiness, model validation.		
44	Xu X et al. 2024. *Repurposing disulfiram with CuET nanocrystals for inflammatory bowel diseases*	67	To investigate nanocrystal-enhanced disulfiram repurposing for inflammatory bowel disease.	Formulation dependency, delivery, safety, mechanism-specific validation.	Formulation innovation can unlock repurposing potential but creates new development requirements.	Scientific/Clinical; Practical
45	Pirola C et al. 2025. *Repurposing daclatasvir for MASLD therapy*	68	To discuss daclatasvir repurposing for MASLD.	Efficacy uncertainty, metabolic-disease complexity, safety validation.	Repurposing for MASLD requires disease-specific evidence and long-term benefit–risk assessment.	Scientific/Clinical
46	Gamal H et al. 2025. *AI, in silico, and CRISPR technologies in cancer repurposing*	69	To review advanced technologies for identifying repurposed cancer therapies.	Validation gaps, model complexity, experimental confirmation, translational uncertainty.	AI/in silico/CRISPR methods expand discovery but require mechanistic and clinical validation.	Practical; Scientific/Clinical
47	Wang R et al. 2025. *Graph neural networks driven acceleration in drug discovery*	33	To review GNNs in drug discovery.	Data sparsity, interpretability, robustness and benchmark limitations.	GNNs accelerate prediction but remain dependent on high-quality data and external validation.	Practical; Scientific/Clinical
48	Scholte M et al. 2025. *Revising EU pharmaceutical legislation: will it foster drug repurposing?*	43	To assess whether EU pharmaceutical legislation reform may support repurposing.	Legislative uncertainty, incentive gaps, data protection/exclusivity issues.	Regulatory reform may improve repurposing only if incentives and evidence pathways are aligned.	Regulatory/IP; Financial/Business
49	Bhat VN et al. 2025. *AI in the pharmaceutical sector*	70	To review AI intervention in drug discovery and manufacturing.	Implementation, data quality, explainability, regulatory trust.	AI may improve pharmaceutical processes but requires governance and validation for repurposing relevance.	Practical; Regulatory/IP
50	Singh A. 2024. *Artificial intelligence for drug repurposing against infectious diseases*	28	To review AI-enabled repurposing for infectious diseases.	Pathogen variability, resistance, limited datasets, clinical validation gaps.	AI may accelerate infectious-disease repurposing but must be resistance-aware and clinically validated.	Practical; Scientific/Clinical
51	Vora N et al. 2025. *Artificial intelligence and multi-omics in drug discovery*	30	To review deep learning and multi-omics approaches.	Omics integration, heterogeneity, model generalizability, validation.	AI and multi-omics provide powerful discovery tools but require integrated, validated data ecosystems.	Practical; Scientific/Clinical

52	Jarallah S et al. 2025. *Artificial intelligence revolution in drug discovery*	34	To review AI-driven innovation in drug discovery.	Black-box modelling, implementation readiness, governance and validation limits.	AI represents a paradigm shift in discovery, but translational use requires explainability and evidence standards.	Practical; Regulatory/IP
53	Recino A et al. 2025. *Therapeutic innovation in drug repurposing: Challenges and opportunities*	71	To review opportunities and challenges in therapeutic repurposing.	Scientific, regulatory, financial and implementation barriers.	Repurposing success depends on integrated management of cross-domain barriers.	Scientific/Clinical; Regulatory/IP; Financial/Business
54	Abu-Zahra T et al. 2024. *How health technology assessment can help to address challenges in drug repurposing*	72	To propose an HTA framework for repurposed medicines.	Evidence appraisal, reimbursement, value recognition, payer acceptance.	HTA can help align clinical evidence, value assessment and reimbursement for repurposed drugs.	Financial/Business; Regulatory/IP
55	Makarani N et al. 2025. *Leishmaniasis: diagnosis, maladies, drug repurposing and way forward*	73	To review leishmaniasis and repurposing opportunities.	Neglected-disease funding gaps, evidence scarcity, access barriers, resistance.	Repurposing in neglected diseases requires sustained investment and implementation-focused validation.	Scientific/Clinical; Financial/Business
56	Alipourgivi F et al. 2025. *Cracking PRMT5: Mechanistic insights, clinical advances, and AI-driven strategies*	74	To review PRMT5 mechanisms and AI-driven therapeutic strategies.	Mechanistic uncertainty, clinical translation, validation of AI-prioritized strategies.	Mechanism-informed and AI-supported strategies may aid repurposing but require experimental confirmation.	Scientific/Clinical; Practical
57	Kuswanto W et al. 2024. *Repurposing drugs for the treatment of osteoarthritis*	75	To review repurposed drugs for osteoarthritis.	Chronic treatment duration, slow endpoints, weak disease-modification evidence.	Osteoarthritis repurposing requires long-term evidence, appropriate endpoints and disease-modifying validation.	Scientific/Clinical
58	Wei S et al. 2025. *Knowledge graphs for drug repurposing*	76	To review knowledge-graph approaches from classical ML to GNNs.	Incomplete graphs, biased data, interpretability, benchmark inconsistency.	Knowledge graphs improve inference but remain limited by data quality and validation requirements.	Practical; Scientific/Clinical
59	Petykó Z et al. 2024. *Policy barriers to using existing pharmaceuticals in new indications*	77	To systematically review policy barriers to repurposing existing medicines.	Legislative gaps, MAH dependency, weak incentives, formal approval barriers.	Policy reform is needed to convert off-label or academic evidence into approved new indications.	Regulatory/IP; Financial/Business
60	Bhat AR et al. 2025. *Artificial intelligence in drug design and discovery*	78	To comprehensively review AI in drug design and discovery.	Bias, overfitting, explainability, validation and implementation limits.	AI supports discovery but does not remove translational, regulatory or validation requirements.	Practical; Scientific/Clinical

61	Alaeddini Z et al. 2025. *Graph-based knowledge distillation framework for drug repurposing*	79	To develop a graph-based knowledge distillation framework for repurposing via multi-task learning.	Label sparsity, transferability, generalizability, validation.	Knowledge distillation improves predictive modelling but remains exploratory without biological confirmation.	Practical; Scientific/Clinical
62	Hershan A et al. 2024. *COVID-19 pathogenesis and FDA-approved repurposed antivirals*	26	To review FDA-approved repurposed antivirals for SARS-CoV-2.	Emergency evidence limitations, variable efficacy, dose/exposure uncertainty.	COVID-19 repurposing demonstrates speed but also the risk of insufficiently mature evidence.	Scientific/Clinical; Practical
63	Seo JI et al. 2024. *PK considerations for anthelmintic repurposing: niclosamide*	80	To examine pharmacokinetic barriers using niclosamide as a case study.	Poor solubility, low bioavailability, exposure limitations, formulation needs.	Pharmacokinetic feasibility is a decisive early filter for repurposing candidates.	Scientific/Clinical; Practical
64	Hu S et al. 2025. *Repurposing strategies for protozoan diseases*	81	To explore advances and challenges in protozoan disease repurposing.	Neglected-disease evidence gaps, limited funding, resistance, safety validation.	Protozoan disease repurposing is promising but requires stronger validation and investment.	Scientific/Clinical; Financial/Business
65	Sebastiano M et al. 2024. *Preclinical alternative drug discovery programs for monogenic rare diseases*	82	To compare small-molecule and gene therapy approaches in monogenic rare diseases.	Small populations, limited disease models, feasibility of trials, uncertain benefit–risk.	Rare-disease repurposing requires flexible evidence standards and disease-specific validation.	Scientific/Clinical; Practical
66	Conceição M et al. 2024. *Repurposing doxycycline for Alzheimer's treatment*	83	To assess doxycycline repurposing from a nano-based delivery perspective.	Brain delivery, formulation dependency, tissue targeting, systemic exposure.	CNS repurposing depends strongly on delivery technologies capable of achieving therapeutic brain exposure.	Practical; Scientific/Clinical
67	Gao Z et al. 2024. *Data-driven computational repurposing for opioid use disorder*	84	To review data-driven computational repurposing approaches for opioid use disorder.	EHR bias, confounding, data scarcity, causality limitations.	Data-driven methods support candidate prioritization but require causal and clinical validation.	Practical; Scientific/Clinical
68	Kang H et al. 2025. *LLM-enhanced drug repositioning knowledge extraction*	38	To evaluate LLM-enhanced knowledge extraction for drug repositioning.	Knowledge reliability, hallucination risk, interpretability, validation.	LLMs can accelerate evidence extraction but cannot replace mechanistic or clinical validation.	Practical; Scientific/Clinical

69	Elkady H et al. 2025. *Small-molecule strategies to combat antibiotic resistance*	85	To review small-molecule strategies against antibiotic resistance.	Resistance evolution, toxicity, interactions, formulation and durability challenges.	Anti-resistance repurposing requires adaptive, combination-aware and clinically feasible strategies.	Scientific/Clinical
70	Hassanali Aragh A et al. 2024. *MiRAGE: mining relationships for advanced generative evaluation in drug repositioning*	36	To develop and evaluate a generative framework for mining biomedical relationships to support drug repositioning.	Knowledge incompleteness, relationship extraction reliability, hallucination risk, benchmark uncertainty, external validation limitations.	Generative relationship mining can improve hypothesis generation for repurposing, but its utility depends on reliable knowledge extraction and rigorous biological validation.	Practical; Scientific/Clinical
71	Czechowicz P et al. 2025. *Old drugs, new challenges: reassigning drugs for cancer therapies*	86	To review challenges and opportunities in reassigning existing drugs for cancer treatment.	Tumour heterogeneity, dose optimization, resistance mechanisms, biomarker uncertainty, translational and regulatory barriers.	Drug reassignment in oncology holds promise, but requires biomarker-guided development, optimized exposure strategies, and stronger translational evidence.	Scientific/Clinical; Regulatory/IP
72	van Galen I et al. 2025. *Drug repurposing for osteoarthritis disease modification in the early 21st century*	21	To review repurposed drugs with potential disease-modifying effects in osteoarthritis.	Slow clinical endpoints, weak disease-modification evidence, heterogeneous patient response, long trial durations, translational uncertainty.	Disease-modifying repurposing in osteoarthritis remains promising but depends on robust long-term evidence, improved endpoints, and stratified evaluation strategies.	Scientific/Clinical; Practical
73	De Sousa-Coelho AL et al. 2025. *Drug Repurposing for Targeting Cancer Stem-like Cells in Glioblastoma*	87	To assess drug repurposing strategies targeting cancer stem-like cells in glioblastoma.	Blood–brain barrier penetration, stem-cell heterogeneity, resistance mechanisms, target validation uncertainty, limited clinical translation.	Targeting glioblastoma stem-like cells through repurposing is promising, but requires CNS delivery solutions, mechanism-based candidate selection, and stronger translational validation.	Scientific/Clinical; Practical
